# From Learning to Policy: Adapting National Cancer Control Plans and Implementation to Africa’s Cancer Control Realities

**DOI:** 10.21203/rs.3.rs-6364181/v1

**Published:** 2025-05-13

**Authors:** Nwamaka Lasebikan, Annet Nakaganda, Erinma Elibe, Rebecca Erickson-Hinds, Allison Frank, Paulette Ibeka, Abdulhafiz Zakieh, Mishka K Cira

**Affiliations:** University of Nigeria Teaching Hospital; Uganda Cancer Institute; National Cancer Institute; National Cancer Institute; National Cancer Institute; Clinton Health Access Initiative; National Cancer Institute; National Cancer Institute

## Abstract

**Background:**

The Africa Cancer Research and Control ECHO (Africa Cancer ECHO) convenes cancer researchers, advocates, clinicians, policymakers, and implementers to address cancer control challenges with evidence-based approaches, to inform and respond to a country’s national cancer control planning efforts.

**Aim:**

To conduct a content analysis of the 2020–2021 Africa Cancer ECHO series, mapping discussions to NCCP core elements and identifying gaps, opportunities, and system-level barriers.

**Methods:**

A SWOT analysis of the 2020–2021 Africa Cancer ECHO series was conducted. 19 sessions were recorded, transcribed, and uploaded to Dedoose^®^. Each session was coded using two guiding frameworks: 1) reported strengths, weaknesses, opportunities, and threats (SWOT); and 2) domains of the Core Elements of National Cancer Control Plans (NCCP) Checklist. Also, descriptors were coded to classify session presenter characteristics (i.e., occupation, geographic context). Data was analyzed quantitatively and qualitatively in Excel and Dedoose^®^ (version 9.0.107).

**Results:**

The case presenters came from six countries, and the didactic presenters came from eight countries (two of which overlap with case presenter countries). Coding resulted in a total of 1547 coded excerpts. Of these, 1446 excerpts were coded to the NCCP core elements, and 931 excerpts were coded to SWOT codes. The top NCCP core element category was Service delivery (402 coded excerpts). The top SWOT element was Opportunity (303 coded excerpts), while the remaining three elements were relatively evenly represented. While research and cancer control are the overall focus of the ECHO, the content most referenced through analysis of the coded excerpts identified diagnosis, staging, and screening, service delivery, and finance as the most discussed topic areas. A key enabler and catalyst for change that was most often discussed during the sessions is task shifting, a strategy that involves redistributing tasks among healthcare providers to maximize efficiency and improve access to care.

**Conclusion:**

The content analysis of the 2020–2021 Africa Cancer ECHO series served as a useful evaluative tool to map the topical issues discussed and identify topical areas that need more emphasis. A more flexible, context-aware NCCP framework may be needed to reflect Africa-specific priorities and realities. Enhanced focus on prevention, palliative care, and surveillance is warranted.

## Introduction

The burden of cancer is growing globally, and disproportionately impacts low- and middle-income countries (LMICs). In 2020, it was estimated that the incidence of new cancer cases was 19.3 million globally, and that number is projected to rise to 28.5 million by 2040. Within the same time frame, it is estimated that there will be a 95% increase in new cancer cases in countries categorized as Low on the Human Development Index (HDI) ^1^. One of the tools available to countries to strengthen their health system response to this growing burden in a coordinated way is a National Cancer Control Plan (NCCP) that is evidence-informed and contextually relevant ^2^. An NCCP is a roadmap for countries to lay out, prioritize, and centrally coordinate locally led and implemented initiatives across the cancer continuum. When developed and implemented effectively, national cancer control plans (NCCPs) improve cancer outcomes at the population level. To aid development and evaluation of the plan, a standard reference of NCCP core elements developed by Oar et al was utilized. This is an evidence-based comprehensive checklist that provides a ready-to-use guide to support NCCP development and facilitate internal and external critical appraisal of existing NCCPs for countries.

Typically, the NCCP includes coordination of resources -technical, human, infrastructure, and financial - needed for all areas of cancer control, such as prevention, early detection, diagnosis and treatment, rehabilitation and reintegration, supportive and palliative care, survivorship, service delivery, governance, research, and surveillance ^3^. Multiple challenges in cancer control planning persist in LMICs, including mobilization of resources and collection, collation, and utilization of relevant contextual data; and the development of sustainable and innovative partnerships to implement NCCPs ^4^. Starting in 2012, the National Cancer Institute (NCI) Center for Global Health (CGH), in partnership with UN agencies, cancer centers, and global partners such as the Union for International Cancer Control, convened a series of regional cancer control leadership forums, of which there were two in the Africa region^5^. In 2018, NCI-CGH transitioned the leadership forum program to the Africa Cancer Research and Control ECHO (Africa Cancer ECHO) Program. The ECHO model utilizes an online collaborative learning approach through case-based learning to advance knowledge on a specific topic ^6^. The Africa Cancer ECHO convenes cancer researchers, advocates, clinicians, and policymakers to address topics around cancer control planning. Each session includes a ‘case’ presentation of a cancer control challenge and a ‘didactic’ presentation on evidence-based approaches to address the challenge, to inform and respond to a country’s national cancer control planning efforts.

Since 2019, a volunteer, rotating Steering Committee of multi-disciplinary cancer experts from the Africa region has led the Africa Cancer ECHO. The sessions are organized by volunteer members of the Steering Committee representing research, clinical practice, civil society, and government. Session topics are identified through an annual evaluation survey of the Africa Cancer ECHO distribution list, and past publications have covered the program evaluation of the utility of the ECHO model for advancing cancer control efforts in Africa ^7^. Program evaluation data have shown that participants reported increased self-efficacy and utilization of cancer control concepts and resources, as well as expanded networks and collaborations ^8^.

Each year, the Steering Committee conducts an evaluation survey to measure the extent to which the program achieves its goals, and in 2020, the Steering Committee conducted and published on how the Africa Cancer ECHO responded to the COVID-19 pandemic^9^.

This study aims to evaluate the content and thematic focus of the 2020–2021 Africa Cancer ECHO sessions by mapping discussions to the core elements of National Cancer Control Plans (NCCPs), to identify practical insights, implementation gaps, and system-level barriers in cancer control across African contexts. By linking session content to the NCCP framework, this analysis seeks to inform context-responsive adaptations of cancer control strategies and highlight how regional telementoring models like ECHO can serve as catalysts for policy development, capacity building, and integrated, locally driven cancer care systems.

## Methods

### Setting, Participants, and Procedures

Each year, members of the Africa ECHO identify specific cancer research and control topics of interest through an annual online survey. Thereafter, every Africa Cancer ECHO session was conducted and recorded using Zoom^®^. Participants in the Africa Cancer ECHO program were clinicians, people living with cancer, advocates, policy makers, researchers, and implementers from 25 countries, 19 in Africa and six countries outside of Africa with work in the region (see Fig. 3). Nineteen publicly accessible one-hour sessions were recorded from September 2020 to August 2021 [Appendix A]. Upon joining each session participants are notified that sessions are recorded and shared publicly after the session. Participants give consent to continue participating in the session. The sessions were moderated by a member of the Africa Cancer ECHO Steering Committee; questions and discussions from all participants were actively encouraged in line with the ECHO collaborative learning model. Further descriptions of the ECHO methodology have been detailed in other publications ^11^.

### Data Collection and Analysis

The 19 recorded sessions were transcribed into Microsoft Word (Version 2302, Microsoft Corporation, Santa Rosa, CA, USA) for analysis. The transcribed sessions were uploaded into Dedoose^®^ (version 9.0.107; Los Angeles, CA, USA), a web-based qualitative data analysis software, which was used for coding the content of each transcript. A study advisory group was convened, including five African cancer experts through an open invitation to the Steering Committee members from 2020–2021 (TF, PI, NL, AN, CMS). A study team was convened consisting of six research fellows and staff from the US NCI/CGH (MKC, EE, TE, RE, AF, AZ).

**Figure 1:**
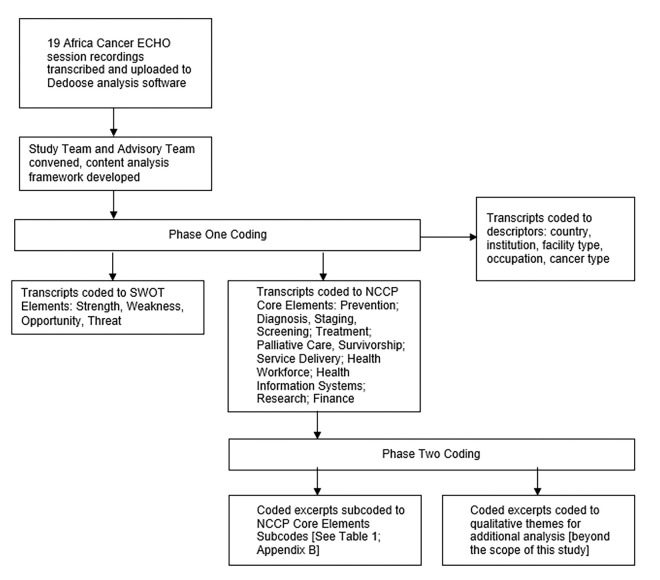
Coding Methodology for Phases 1 and 2

The study team developed a content analysis framework that involved two phases (explained below). The coding protocol was established, and a practice coding session was conducted to ensure coding uniformity throughout the study. In the practice phase, the study team individually coded the same ECHO session in Dedoose. Each piece of coded text was referred to as a coded excerpt. Coding was compared by study team members to ensure inter-reviewer agreement. Once agreement was achieved, the remaining ECHO sessions were assigned to be coded by the study team (one study team member per ECHO session). The study team consulted via Zoom meetings and email for quality control and to address any questions about the data. Figure 1 shows the coding methodology used for both phases of the content analysis.

#### Phase One –

In phase one, excerpts were coded to identify reported strengths, weaknesses, opportunities, and threats (SWOT) about discussed topics, in relation to the core elements of National Cancer Control Plans (NCCPs)^3^: prevention; diagnosis, staging, and screening; treatment; palliative care and survivorship; service delivery; governance; health workforce; health information systems; research; and finance. The Core Elements of NCCPs can be used as a standardized tool to look at the comprehensiveness of an NCCP. This list was organized based on recommendations from the Organization for Economic Cooperation and Development (OECD) for policy makers to address cancer at a population level and was published following the 2018 global review of NCCPs as guidance to countries in national cancer control planning ^12^. Additional coding at this phase identified descriptors of the speaker and topic, such as country, institution, facility type, occupation, and cancer type. See Appendix B for the full codebook.

#### Phase Two –

In this phase, the NCCP core elements codes were further sub-coded into categories as outlined by Oar et al. (e.g., tobacco control and vaccination within Prevention; see Appendix B) (Table 1). During this phase, the coding team also identified qualitative themes that emerged from the coded excerpts. These qualitative themes were recorded as memos within the Dedoose software and saved for future analysis, which is outside the scope of this initial study.

**Figure 2:**
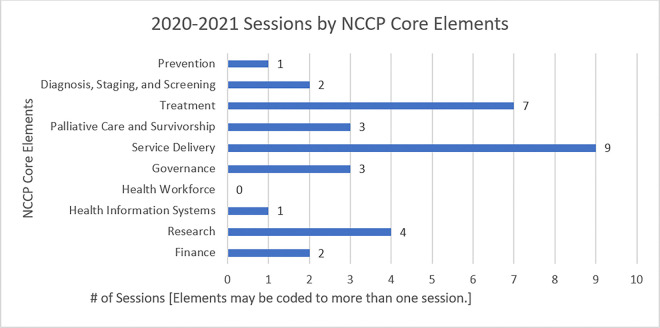
Number of session topics mapped to each NCCP core element (n=19 sessions)

Additionally, each ECHO session topic was coded to the NCCP core elements, as displayed in Fig. 2; session topics could be mapped to more than one core element. Each NCCP core element code was assigned a subcode based on categories within each core element of the NCCP core elements publication^3^.

## Results

**Figure 3:**
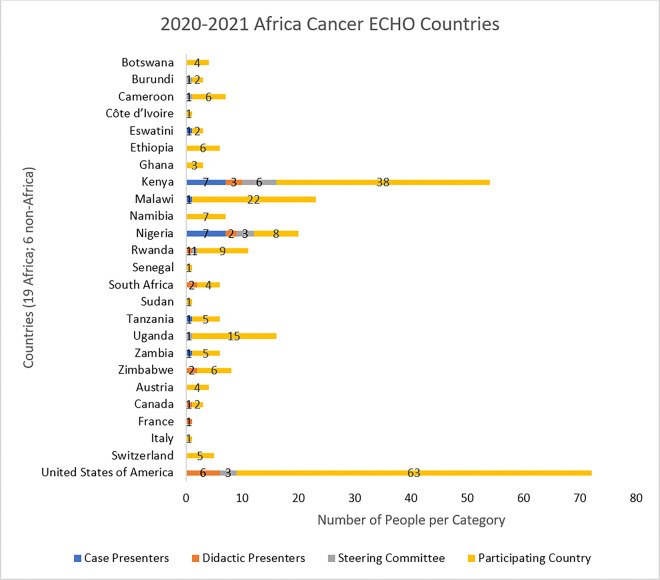
Country representation of the case presenters (6 countries), didactic presenters (8 countries), Steering Committee members (7 countries), and participating countries (25 countries)

**Figure 4:**
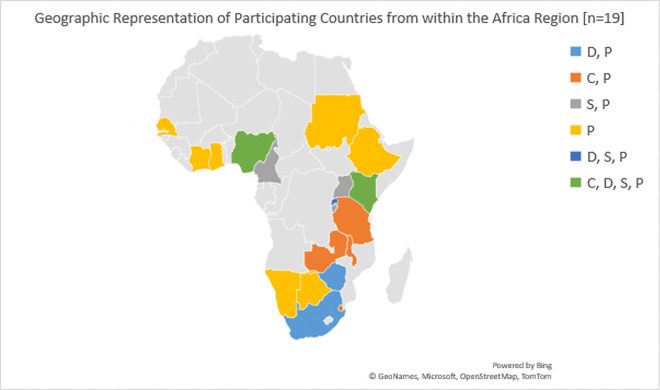
Country representation from within the Africa region: D, P = Didactic Presenter and Participant Country (2 countries); C, P = Case Presenter and Participant Country (4 countries); S, P = Steering Committee Member and Participant Country (3 countries); P = Participant Country (non-presenter, non-Steering Committee – 7 countries); D, S, P = Didactic Presenter, Steering Committee Member and Participant Country (1 country); C, D, S, P = Case Presenter, Didactic Presenter, Steering Committee Member and Participant Country (2 countries).

There were 19 Africa Cancer ECHO sessions [Appendix A], which were held from September 2020 to August 2021. The session case presenters came from six countries and represented six professions. The didactic presenters came from 8 countries and represented six professions. See Fig. 3 for a full list of countries represented by the Steering Committee members and session speakers. See Fig. 4 for the geographic display of case presenters, didactic presenters, and Steering Committee members by country.

The sessions identified through the annual survey of members were: pediatric cancer, prostate cancer, access to medicines, radiation therapy, clinical practice guidelines, palliative care, survivorship, patient-centeredness (patient navigation, vulnerable populations), research (patient engagement, partnerships, dissemination and implementation research), and system strengthening (mHealth and telehealth, costing strategies, integration, measuring impact).

Coding the transcripts resulted in a total of 1,547 coded excerpts. Of these, 1,446 excerpts were coded to align with the NCCP core elements, and 931 excerpts were coded to SWOT codes. The top NCCP core element coded categories were service delivery (402 coded excerpts), diagnosis, staging, screening (173 coded excerpts), and finance (158 coded excerpts).

When sessions were coded based on core NCCP elements, Service delivery had the highest numbers followed by treatment and research, representing the interest and priority of these NCCP core elements within the community.

Each NCCP core element included a set of excerpts that did not fit into the sub coded categories and there were 511 (33%) identified codes transcribed during the sessions that did not align to NCCP core elements framework currently in use that guide planning, evaluation and implementation in different countries indicating potential limitations in capturing priority areas within the region. These are listed as uncoded excerpts, and the full set of figures showing subcodes by NCCP core element is in Appendix D.

**Figure 5:**
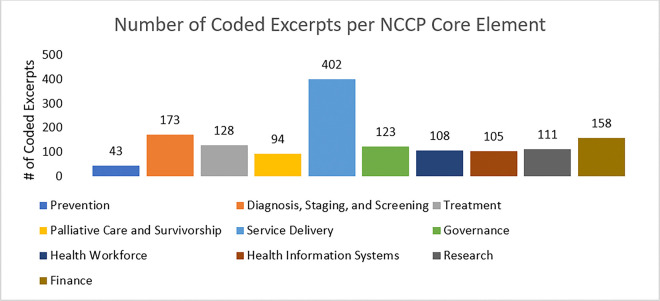
Number of coded excerpts per NCCP core element [n=1,446]

See Fig. 5 for a full breakdown of coded excerpts by NCCP core element [n = 1,446]

The top SWOT element for NCCP core elements was Opportunity, with 303 coded excerpts for Service delivery. Other opportunities were also noted in Health Information System, Governance and Finance, highlighting potential areas for development. Similarly significant weaknesses were also coded in Service Delivery, Treatment and Diagnosis, Staging and Screening, signifying gaps and challenges with these core elements noted by the community. Diagnosis, Staging, Screening, and Finance core elements had almost similar codes in opportunity and threats, signifying potential areas for development and barriers to implementation and sustainability.

**Figure 6:**
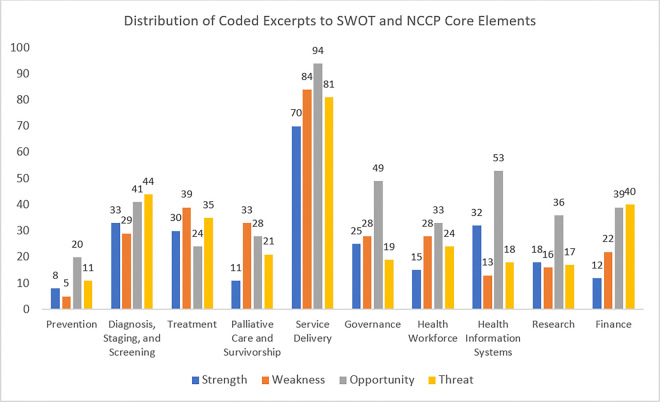
Distribution of coded excerpts to SWOT and NCCP core elements

For the distribution of SWOT analysis of coded excerpts of NCCP core elements, see Fig. 6.

Appendix C lists the number of coded excerpts separately by SWOT elements and NCCP core elements.

Further sub-coding of the NCCP Core elements: noted that the top subcode for Prevention core element was vaccination (Human papillomavirus (HPV), Hepatitis B virus (HBV)) with 19 coded excerpts. Cancer awareness and early detection were the highest subcodes in the Diagnosis, Screening and staging core element, while paediatric cancer, treatment guidelines, essential medicines, and multidisciplinary team care were subcoded under treatment.

Palliative workforce training, survivorship care and survivorship care plan, health workforce training, and patient navigation were most commonly coded for Palliative care and healthcare workforce. Under the Finance subcode, costing, financial resources, and financial protection for patients (financial toxicity) were the common codes seen.

And the top subcode for the Governance core element was monitoring and evaluation (21 coded excerpts). The full breakdown of the number of excerpted codes by NCCP core elements and subcodes is shown in Table 1

A few examples of text cross-coded to SWOT and NCCP core elements include:

*Strength* and *Diagnosis, Staging, and Screening*: description of the use of technology to reduce time to diagnosis.

*Weakness* and *Service Delivery*: Description of how the lack of information and knowledge among patients and caregivers leads to high mortality rates.

*Opportunity* and *Prevention*: Description of the idea of integrating scientific research with social services to reduce HIV and cervical cancer risk factors.

*Threat* and *Health Workforce*: description of the shortage of specialists in hospitals which has the potential to lead to long wait times and lack of access to care for patients.

## Discussion and Conclusion

In this paper, we describe the results of a content analysis of the 19 Africa Cancer ECHO sessions held between September 2020 and August 2021. The Africa Cancer ECHO activities are designed to address key elements across the core tenets of the NCCP continuum - prevention and early detection, clinical management, cancer support services, and palliative care through non-traditional means. The Extension for Community Healthcare Outcomes (ECHO) has been validated as a model suitable for tele-mending and continuous professional development at a global scale. Despite the ongoing COVID 19 pandemic in the study period (2020–2021), participation in the ECHO remained robust.

The faculty for the year-long series was diverse, bringing expertise from across different professions, highlighting the need for multidisciplinary involvement in cancer control. Similarly, more than half of the speakers for didactic presentations were from the African continent, reflecting the importance of centering regional leadership for decisions based on solid and context-specific knowledge to address regional problems. The core elements of a National Cancer Control Plan (NCCP) include prevention and population-level driven policies, surveillance and monitoring to inform cancer control, treatment and care services, cancer management and support for people diagnosed with cancer, and integration of palliative care into the cancer plan. Our study revealed that numerous sessions focused on opportunities to reinforce crucial components of cancer control programs. While research and cancer control are the overall focus of the ECHO, the content most referenced through analysis of the coded excerpts identified diagnosis, staging, and screening, service delivery, and finance as the most discussed topic areas. These three combined NCCP core elements had significantly more focus and could represent elements with the most immediate opportunities and threats towards the continents’ cancer control efforts. The content analysis also identified systemic and structural challenges as well as resource and infrastructure limitations in coverage of key NCCP core elements, including prevention, screening, diagnosis, staging, treatment, palliative care health workforce, finance and health information systems; all of which need to be addressed to build a successful cancer control program. By mapping the session topics from the Africa Cancer ECHO series onto the cancer control continuum, we have illustrated how facets of individual discussions are connected to broader cancer control, providing a unique opportunity to explore potential linkages between various stakeholders and their activities. Additionally, the role of technology for tele mentoring has multi-sector benefits, including potentially unearthing additional linkages to strengthen cancer control efforts on the continent.

With regards to the specific NCCP core elements discussed, a key enabler and catalyst for change that was most often mentioned or discussed during the sessions is task shifting, a strategy that involves redistributing tasks among healthcare providers to maximize efficiency and improve access to care. While task shifting can help address the challenge of access to cancer care in Africa, it should be implemented within a comprehensive healthcare system that also focuses on strengthening infrastructure, increasing funding, and improving cancer surveillance and data collection^13,14^. In Rwanda task shifting has been employed to enhance breast cancer care through the integration of community health workers (CHWs) and nurses in the healthcare delivery system. The initiative involved training CHWs and nurses to perform basic breast cancer screenings, education, and referrals. This approach aimed to address the shortage of oncologists and specialized healthcare providers in the country^15^

As the continent continues to struggle with the heavy burden associated with late-stage presentation and access to diagnosis and care, more discussion points focused on prevention and early detection to identify barriers and facilitators to early detection and prompt treatment.. Unsurprisingly, vaccination against HBV and HPV was represented within the coded excerpts and correlated well with the heavy burden of malignancies associated with these infections on the continent ^16^. To address the burden of malignancies associated with HBV and HPV infections, vaccination programs against these diseases are crucial elements in cancer control efforts. In addition to vaccination, cancer control efforts should include early detection and treatment of infections that may lead to cancer, which will in turn reduce incidence, mortality, and morbidity of hepatic and cervical cancer on the continent. These important and relevant topics should be included in future Africa Cancer ECHO sessions. Due to less emphasis being placed on topics concerning prevention in 2020–2021, the Africa Cancer ECHO leadership, starting in 2022, has taken measures to ensure more equal coverage of each NCCP core element by assigning each of the ten NCCP core elements to a specific month in the series for the year.

System level challenges focusing on treatment guidelines, access to essential medicines, multidisciplinary team care, palliative workforce training, survivorship care and survivorship care plan; health workforce training and patient navigation as well as costing, financial resources and financial protection for patients (financial toxicity) are opportunities for development of innovative locally relevant, transformative and sustainable strategies that can improve the often flawed and disjointed health systems found in many African nations. ^17,18,19^

There are some examples of ongoing regional partnerships to address system-level weaknesses such as the development of context and resource appropriate guidelines ^20,21^for standardizing care, surgical oncology training led by cross-regional collaborations ^22,23^, coalition-building to address lack of access to medicines and infrastructure for safe delivery of chemotherapy ^24^, and development of sustainable train-the-trainer programs ^25,26^. Other potential strategies to address these weaknesses include investments in subspecialty education and training for healthcare professionals, as well as quality improvement programs. It is important to note that access to palliative care was recognized as a human right by the World Health Organization ^27,28^ and therefore warrants integration into national health policies and action plans. The Africa Cancer ECHO should continue to incorporate knowledge sharing about advances to address system-level weaknesses, especially those that are relevant and feasible to adapt to the diverse settings represented by session participants.

There may be a need to tailor the current NCCP core element framework to be responsive to the African context, considering that about two-thirds of the coded excerpts from phase one could not be captured with the current framework.

There were several limitations to this study. Two limitations that are beyond the control of the study team are of internet connectivity, which can sometimes be a limitation to participants, as well as competing priorities of the participants. Additional limitations specific to this study include the following: First, each transcript was coded by one reviewer, and individual interpretation and coding practices may have varied by reviewer. Second, much of the transcribed content did not fit into the SWOT framework or NCCP core elements (i.e., uncoded excerpts), which may have resulted in relevant information being omitted from our analyses. Third, while further qualitative analysis will provide insights into the types of strengths, weaknesses, opportunities, and threats in the cancer control systems in Africa, the content used for these analyses is based on the individual perspectives of the Africa Cancer ECHO participants and may not reflect broader issues and challenges. Last, the content we analyzed was based on the topics selected for the 2020–2021 Africa Cancer ECHO series and may not have represented the cancer control topics of most relevance to the whole region. While the Africa Cancer ECHO aims to reach participants from around the continent, in our current mapping, the participants came from 19 countries in the Africa region, while the case/didactic speakers came from nine countries. The Africa Cancer ECHO Steering Committee would need to consider ways to expand the diversity of representation of voices to describe country-level context from other participating countries. With a significant proportion of African countries not participating in the 2020–2021 Africa Cancer ECHO series, more should be done to understand the limitations to participation (including language barriers, access to internet, knowledge about the program, etc.). Another limitation could be time constraint due to competing work-related interests,s considering the limited skilled workforce and the heavy work burden these professionals have to contend with. Given the unique nature of this community of practice for regular dialogue in real time on issues related to cancer research and cancer control in Africa, this community should provide resources and connections needed in other countries in the region.

In conclusion, the content analysis of the 2020–2021 Africa Cancer ECHO series served as a useful evaluative tool by providing greater insight into the diversity of topical issues that the Africa Cancer ECHO session participants prioritized for discussion. Further analysis is warranted to explore what is discussed around the most frequently addressed NCCP core elements. At the same time, this analysis identified key NCCP core elements that were given less emphasis, and the limited number of countries represented through speaking roles in sessions. This information can be used to guide future session development to ensure broader inclusion of topics and geographic representation in the Africa Cancer ECHO in years to come.

## Figures and Tables

**Table 1:** Full breakdown of the number of excerpts coded to NCCP core elements and subcodes

NCCP Core Element	Subcode	# of Subcoded Excerpts
Prevention (43 coded excerpts)	Vaccination (Human papillomavirus, Hepatitis B virus)	19
	Risk factors (environment, occupational)	6
	Cancer awareness/public education (Prevention)	5
	Disease risk factors (Hepatitis C, HIV)	5
	Tobacco control	4
	Modifiable risk factors (tobacco/alcohol use, physical activity, nutrition)	1
	Uncoded excerpts	3
Diagnosis, Staging, and Screening (173 coded excerpts)	Cancer awareness/public education (early detection)	28
	Pathology reporting/training/access	23
	Breast cancer screening/early detection	17
	Cervical cancer screening/early detection	10
	Primary care/primary care training (diagnosis)	9
	Cancer-specific early detection (colon, oral, childhood)	3
	Cancer diagnosis/staging guidelines	3
	Uncoded excerpts	80
Treatment (128 coded excerpts)	Pediatric cancer care/guidelines	28
	Treatment guidelines/protocols	23
	Essential medicines list/procurement/supply chain	16
	Radiation oncology/training	15
	Multi-disciplinary teams/coordination of care	14
	Devices/machinery/technology	7
	Guidelines implementation/quality control	5
	National guidelines (cancer-specific)	3
	Surgical oncology/training	1
	Uncoded excerpts	16
Palliative Care and Survivorship (95 coded excerpts)	Survivorship/follow-up/long-term care	42
	Palliative care workforce/training	28
	Psycho-social/non-physical care	8
	Primary care/primary care training (palliative/supportive care)	6
	Family/caregiver support	5
	Home-based/community-based care	3
	Pain management	0
	Rehabilitation	0
	Uncoded excerpts	3
Service Delivery (402 coded excerpts)	Vulnerable populations	65
	Task shifting/decentralization	56
	Community/family engagement and empowerment	30
	Chemotherapy patient/staff safety	6
	Uncoded excerpts	245
Governance (123 coded excerpts)	Monitoring and evaluation	21
	Accountability frameworks	10
	Participatory governance	7
	Patient/patient group involvement	2
	Leadership in cancer control	1
	Uncoded excerpts	82
Health workforce (108 coded excerpts)	Health workforce strategy	41
	Patient navigation (health workforce)	41
	Oncology nursing	2
	Pharmacy	0
	Uncoded excerpts	24
Health information systems (105 coded excerpts)	Cancer clinical care indicators	36
	Cancer registry/reports	31
	Hospital-based registry	0
	Uncoded excerpts	38
Research (111 coded excerpts)	Cancer research plan	80
	Cancer research funding	14
	Uncoded excerpts	17
Finance (158 coded excerpts)	Costing	71
	Financial resources	50
	Financial protection for patients (financial toxicity)	18
	Foreign aid	8
	Multisectoral partnership	5
	Sub-national/state level	3
	Uncoded excerpts	3

## Data Availability

The data that support the findings of this study are available from Africa Cancer Research and Control ECHO, but cannot be made publicly available. The data are, however, available from the authors upon reasonable request.

## References

[1] Sung H, Ferlay J, Siegel RL, : Global Cancer Statistics 2020: GLOBOCAN Estimates of Incidence and Mortality Worldwide for 36 Cancers in 185 Countries. CA Cancer J Clin 71:209–249, 2021 33538338 10.3322/caac.21660

[2] Torode JS, Tittenbrun Z, Romero Y, : Ten Years of the International Cancer Control Partnership: Promoting National Cancer Control Plans to Shape the Health System Response for Cancer Control. JCO Glob Oncol 9:e2200232, 2023 36630665 10.1200/GO.22.00232 PMC10166518

[3] Oar A, Moraes FY, Romero Y, : Core elements of national cancer control plans: a tool to support plan development and review. Lancet Oncol 20:e645–e652, 2019 31674323 10.1016/S1470-2045(19)30404-8

[4] Duncan K, Cira MK, Barango P, : Challenges and opportunities in the creation and implementation of cancer-control plans in Africa. Ecancermedicalscience 13:938, 2019 31552111 10.3332/ecancer.2019.938 PMC6722107

[5] Duncan K, Cira M, Ng’ang’a A: Use of telementoring to advance cancer control: The 2018 Africa Cancer Research and Control ECHO^®^ Programme Cancer Control:69–74, 2019

[6] Arora S, Thornton K, Murata G, : Outcomes of treatment for hepatitis C virus infection by primary care providers. N Engl J Med 364:2199–207, 2011 21631316 10.1056/NEJMoa1009370 PMC3820419

[7] Nakaganda A, Cira MK, Abdella K, : Expanding best practices for implementing evidence-based cancer control strategies in Africa: The 2019–2020 Africa Cancer Research and Control ECHO Program. J Cancer Policy 28:100286, 2021 35559915 10.1016/j.jcpo.2021.100286

[8] Lasebikan N, Nakaganda A, Fadelu T, : Demonstration of Impact Through the Evaluation of the 2020–2021 Africa Cancer Research and Control ECHO. JCO Global Oncology 8:37–37, 2022

[9] Nakaganda A, Lasebikan N, Garton EM, : How COVID-19 exposed pre-existing roadblocks for cancer control in Africa: strategies, lessons and recommendations from the 2019–2020 Africa Cancer Research and Control ECHO. Ecancermedicalscience 17:1516, 2023 37113714 10.3332/ecancer.2022.1516 PMC10129397

[10] Teoli D, Sanvictores T, An J: SWOT Analysis. StatPearls [Internet], Treasure Island (FL): StatPearls Publishing, https://www.ncbi.nlm.nih.gov/books/NBK537302/, 2023 January 30725987

[11] Chicoine G, Côté J, Pepin J, : Effectiveness and experiences of the Extension for Community Healthcare Outcomes (ECHO) Model in developing competencies among healthcare professionals: a mixed methods systematic review protocol. Syst Rev 10:313, 2021 34911579 10.1186/s13643-021-01832-0 PMC8675457

[12] Romero Y, Trapani D, Johnson S, : National cancer control plans: a global analysis. Lancet Oncol 19:e546–e555, 2018 30268693 10.1016/S1470-2045(18)30681-8

[13] Joshi R, Alim M, Kengne AP, : Task shifting for non-communicable disease management in low and middle income countries--a systematic review. PLoS One 9:e103754, 2014 25121789 10.1371/journal.pone.0103754 PMC4133198

[14] O’Donovan J, Newcomb A, MacRae MC, Community health workers and early detection of breast cancer in low-income and middle-income countries: a systematic scoping review of the literature. BMJ Global Health 2020;5:e002466. doi:10.1136/bmjgh-2020-002466 PMC7228495 32409331

[15] Pace LE, Dusengimana J-MV, Keating NL, Impact of breast cancer early detection training on Rwandan health workers’ knowledge and skills. J Glob Oncol 2018;4:1–10. 10.1200/JGO.17.00098 PMC6223427 30241228

[16] Ngwa W, Addai BW, Adewole I, : Cancer in sub-Saharan Africa: a Lancet Oncology Commission. 10.1016/S1470-2045(21)00720-8 PMC9393090 35550267

[17] Malakoane B, Heunis JC, Chikobvu P, Kigozi NG, Kruger WH. Public health system challenges in the Free State, South Africa: A situation appraisal to inform health system strengthening. BMC Health Services Research. 2020 Dec;20:1–4. springer.com 10.1186/s12913-019-4862-y PMC6979387 31973740

[18] Eruaga MA, Itua EO, Bature JT. Enhancing medication quality control in Nigeria: a comprehensive analysis of regulatory challenges and solutions. International Medical Science Research Journal. 2024 Mar 17;4(3):284–94. fepbl.com

[19] Cortes J, Perez‐García JM, Llombart‐Cussac A, Curigliano G, El Saghir NS, Cardoso F, Barrios CH, Wagle S, Roman J, Harbeck N, Eniu A. Enhancing global access to cancer medicines. CA: a cancer journal for clinicians. 2020 Mar;70(2):105–24. wiley.com Lancet Oncol 23:e251-e312, 2022 32068901 10.3322/caac.21597

[20] Mutebi M, Adewole I, Orem J, : Toward Optimization of Cancer Care in Sub-Saharan Africa: Development of National Comprehensive Cancer Network Harmonized Guidelines for Sub-Saharan Africa. JCO Glob Oncol 6:1412–1418, 2020 32970487 10.1200/GO.20.00091 PMC7529540

[21] DeBoer RJ, Ndumbalo J, Meena S, : Development of a theory-driven implementation strategy for cancer management guidelines in sub-Saharan Africa. Implement Sci Commun 1:24, 2020 32885183 10.1186/s43058-020-00007-7 PMC7427872

[22] Ezeome ER, Ekenze SO, Ugwumba F, : Surgical training in resource-limited countries: moving from the body to the bench--experiences from the basic surgical skills workshop in Enugu, Nigeria. Trop Doct 39:93–7, 2009 19299292 10.1258/td.2009.080422

[23] Parker AS, Steffes BC, Hill K, : An Online, Modular Curriculum Enhances Surgical Education and Improves Learning Outcomes in East, Central, and Southern Africa: A Mixed-Methods Study. Ann Surg Open 3:e140, 2022 37600087 10.1097/AS9.0000000000000140 PMC10431403

[24] Dare AJ, Bayle A, Hatoqai A, : Ensuring Global Access to Cancer Medicines: A Generational Call to Action. Cancer Discov 13:269–274, 2023 36734325 10.1158/2159-8290.CD-22-1372

[25] Mormina M, Pinder S: A conceptual framework for training of trainers (ToT) interventions in global health. Global Health 14:100, 2018 30348183 10.1186/s12992-018-0420-3 PMC6198384

[26] Snowden B, Lahiri S, Dutton R, : Achieving and Sustaining Change Through Capacity Building Train-the-Trainer Health Initiatives in Low- and Middle-Income Countries: A Systematic Review. J Contin Educ Health Prof 43:96–103, 2023 36215155 10.1097/CEH.0000000000000458

[27] Frissen AR, Burgers S, van der Zwan JM, : Experiences of healthcare professionals with support for mesothelioma patients and their relatives: Identified gaps and improvements for care. Eur J Cancer Care (Engl) 30:e13509, 2021 34498770 10.1111/ecc.13509

[28] Qanungo S, Calvo-Schimmel A, McGue S, : Barriers, Facilitators and Recommended Strategies for Implementing a Home-Based Palliative Care Intervention in Kolkata, India. Am J Hosp Palliat Care 38:572–582, 2021 33167661 10.1177/1049909120969127

